# Supplementary material for: Symptom profiles in lung cancer survivors: A latent class approach
Source: PLoS One. 2025 Oct 31;20(10):e0309272. doi: 10.1371/journal.pone.0309272 (PMC12578159; doi:10.1371/journal.pone.0309272)
Supplement: Attachment — Submitted filename: Responses to Reviewers_Lee 27FEB25.docx [file pone.0309272.s003.docx]

**Date**:	February 27, 2025

**To**:	Dr. Emily Chenette

Editor-in-Chief of *PLOS One*

**From**:	Lena J. Lee, PhD RN

National Institutes of Health, Clinical Center

**Subject**: Submission of manuscript “Symptom Profiles in Lung Cancer Survivors: A Latent Class Approach” requested revisions.

Please find our revised submission of the manuscript entitled “Symptom Profiles in Lung Cancer Survivors: A Latent Class Approach." We provided our responses to the reviewers’ comments and marked our changes in the body of the manuscript with the yellow-colored font. We confirm that this study has passed Ethical Committee Review. We appreciate the thoughtful and thorough critique by the reviewers and believe this has strengthened the quality of our manuscript.

**Comments from the Reviewers**

| **General Comments** | **Responses to General Comments** |
| --- | --- |
| Please ensure that your manuscript meets PLOS ONE's style requirements, including those for file naming. The PLOS ONE style templates can be found at  <https://journals.plos.org/plosone/s/file?id=wjVg/PLOSOne_formatting_sample_main_body.pdf> and  <https://journals.plos.org/plosone/s/file?id=ba62/PLOSOne_formatting_sample_title_authors_affiliations.pdf> | Thank you for this comment. We have gone ahead and double-checked the formatting of the manuscript with the PLOS ONE style templates. |
| Please include your full ethics statement in the ‘Methods’ section of your manuscript file. In your statement, please include the full name of the IRB or ethics committee who approved or waived your study, as well as whether or not you obtained informed written or verbal consent. If consent was waived for your study, please include this information in your statement as well. | Thank you for your comments. We have added the full ethics statement in the Methods section.  [Methods- Ethical Considerations] Page 8, Lines 184-187  *This study employed a secondary data analysis approach using the MY-Health study. All participants provided informed consent before participation, approved by the institutional review boards of each participating SEER site and Georgetown University, Washington, DC.* |
| Your abstract cannot contain citations. Please only include citations in the body text of the manuscript, and ensure that they remain in ascending numerical order on first mention. | Thank you for this comment. We have double-checked the abstract and did not find any citations within that section. |
| Please ensure that you refer to Figure 2 in your text as, if accepted, production will need this reference to link the reader to the figure. | We have corrected Figure 1 on page 11 to Figure 2. |
| Please review your reference list to ensure that it is complete and correct. If you have cited papers that have been retracted, please include the rationale for doing so in the manuscript text, or remove these references and replace them with relevant current references. Any changes to the reference list should be mentioned in the rebuttal letter that accompanies your revised manuscript. If you need to cite a retracted article, indicate the article’s retracted status in the References list and also include a citation and full reference for the retraction notice. | Thank you for this comment. We have cited one additional paper, which is reflected as Citation #46 both within the body of the paper and within the reference list. |
| **Comments from Reviewer 1** | **Responses to Reviewer 1** |
| The paper addresses an important and current topic in cancer survivorship and care. However, the cross-sectional nature of the study severely limits the conclusions that can be drawn from the work. A second limitation is that the assessments all used the PROMIS system which does not enable comparison with other work in the symptom cluster area of cancer: it is not clear what these assessments actually measure or what their scores indicate in terms of severity or other dimensions. No explanation is given. | Thank you for this comment. We have addressed the limitation of the cross-sectional design in another comment for limitations. In addition, we apologize for not being clearer about the features of PROMIS measures, such as their scoring interpretation and compatibility with other work. To address the reviewers’ concerns, we have provided more details on the PROMIS measures to the Methods section.  [Methods-Measures] Page 6, Lines 137-143  *The PROMIS measures are scored on a five-point Likert-type scale, rating from 1 (never) to 5 (always), with higher scores indicating greater symptom severity. The PROMIS are calibrated and standardized to a T-score metric, with a mean of 50 and standard deviation of 10 centered on the general U.S population. The PROMIS enables comparisons across studies using other instruments that measure the same health concept, for example, the Center for Epidemiologic Studies Depression Scale (CES-D) and the Functional Assessment of Chronic Illness Therapy (FACIT)-Fatigue.* |
| There is no information on how the (presumed) subsample of lung cancer patients were derived from the SEER MY-health study, again making interpretation of the results difficult. | Thank you for the comments. We apologize for not providing sufficient detail on how the subsample of lung cancer patients were derived from the SEER MY-Health study. We have added the following sentence.  [Methods-Samples and Setting] Page 5, Lines 108-116  *Participants were eligible for the study if they (a) were aged 21-84 years at the time of initial diagnosis of one of seven types of cancer (i.e., female breast cancer, prostate cancer, colorectal cancer, non-small cell lung cancer, non-Hodgkin lymphoma, uterine cancer, and cervical cancer) and (b) had the ability to read and speak English, Spanish, or Mandarin. The survey was completed by 5,506 people with cancer. For this analysis, the authors restricted eligibility to individuals diagnosed with non-small cell lung cancer within the past 6-13 months. Of 722 patients with lung cancer, a total of 526 participants were included following the exclusion of 35 participants diagnosed more than 13 months prior to the study and 161 participants who had died during the survey period.* |
| The results need to be more clearly presented. There is no information provided on data coding so we do not know what the significant relationship with income means, for example. The referent is > $60K and the ORs are all positive. I’m assuming that the lower income groups have the higher ORs, but it is not discernable from the way the data are presented. | Thank you for the comments. We apologize that we were not clearer about the data coding. We clarified the statement.  [Methods- Measures] Pages 5-6, Lines 119-131  *Influential Factors. Demographic and socioeconomic factors included age at diagnosis, sex, race (white, non-white [multiple, black, Asian, American Indian or Alaska Native, Asian Hawaiian or Pacific Islander, other]), ethnicity (Hispanic, non-Hispanic), marital status (married [married, living with partner], not married [never married, separated, divorced, widowed]), education level (≤ high school [< high school graduate, high school graduate], ≥ some college [some college, college degree, graduate degree]), employment status (working [working full time, full time homemaker, part time, student], not working [retired, disability, unemployed]), annual income (< $60K [less than $10,000, $10,000 to $59,999], ≥ $60K [$60,000 to $99,999, $100,000 to $199,999, $200,000 or more]), and insurance status (private [private, private and government], not private [government only, no insurance]) . Clinical factors included smoking history (yes, no), cancer stage (I, II, III, IV) , cancer treatment history (no treatment, surgery only, chemotherapy only, radiation therapy only, combination therapy) , and the number of self-reported comorbidities (0, ≥ 1).* |
| A further problem is that the data are all from the USA, which is an outlier in terms of the costs of the health care system combined with high levels of social problems, and as such does not represent the majority of nations globally. As the social factors are identified as predictors, the results are therefore of limited utility beyond their national boundaries. | Thank you for your thoughtful comments. To address the reviewer’s concerns, we have provided more details on how well the data reflect demographic of the US population and added the language regarding the lack of generalization to global populations to the limitation section.  [Discussion- Limitation] Page 19, Lines 389-395  *In that vein, we note that the SEER areas cover approximately 30% of the US population and aims to reflect the diverse demographics of the US population. While the registry areas are not considered statistically representative of the U.S. population, studies have shown no significant differences in demographics (age, race, or sex).^46^ However, this U.S. based data analysis cannot be generalized to symptom cluster trends on a global level due to the limitations posed by the U.S. being an outlier when it comes to healthcare costs and associated social issues.* |
| Because the data are cross-sectional, the authors are unable to decompose the causal directions for these symptom clusters. It is already well-known that symptoms cluster in cancers. Sleep disturbance may be due to cancer deregulation of circadian function, to anxiety, to pain or, as is often the case, may pre-date the diagnosis and be a lifelong problem. Ditto with depression and we do not know if the pain described is due to cancer, a late-effect of treatment or arthritis, LBP, or other benign conditions. Similarly, low income households are known to report much higher prevalence of depression independent of physical health. | Thank you for your comment. First, we have addressed the limitation of the cross-sectional design in another comment for limitations. In addition, we agree that there is an accumulated body of evidence on symptom clusters in cancer. However, we found that symptom clusters in patients with lung cancer and their survivors are not well understood compared to other types of cancers. Therefore, we have further explained the point, and added following statements on the lack of evidence regarding symptom clusters in lung cancer patients, along with further details on the causal directions of the symptom clusters in the Discussion Section.  [Discussion] Page 19, Lines 379-388  *There is limited evidence regarding symptom clusters in lung cancer survivors, while the notion that symptoms in survivors of other types of cancer, such as breast cancer, gastrointestinal cancer, occur in a cluster orientation is well-known and documented. Given the prevalence of lung cancer, especially in the United States, it is important to highlight not only factors influencing symptom cluster membership for lung cancer survivors, but also how these symptoms impact physical and social performance/function for those impacted. It is also important to note that the cross-sectional nature of this study makes it difficult to identify the causal directions for the highlighted symptom clusters, as sleep, fatigue, pain, and depression can be attributed to a plethora of factors such as treatment, underlying disease, and socioeconomic factors.* |
| **Comments from Reviewer 2** | **Responses to Reviewer 2** |
| Methodology: The authors state that the “Details of the study design, study procedures, and participant descriptions of MY-Health study have been reported elsewhere.” I would request to include the percentage of the 526 individuals represented in the original study and, furthermore, how many lung cancer survivors are estimated in the specific registries and how representative are the included registries considering all registries in SEER database. (page 5) | Thank you for the comments. We apologize for not providing sufficient detail on how the subsample of lung cancer patients were derived from the SEER MY-Health study. We have added the following sentence.  [Methods] Page 5, Lines 108-116  *Participants were eligible for the study if they (a) were aged 21-84 years at the time of initial diagnosis of one of seven types of cancer (i.e., female breast cancer, prostate cancer, colorectal cancer, non-small cell lung cancer, non-Hodgkin lymphoma, uterine cancer, and cervical cancer) and (b) had the ability to read and speak English, Spanish, or Mandarin. The survey was completed by 5,506 people with cancer. For this analysis, the authors restricted eligibility to individuals diagnosed with non-small cell lung cancer within the past 6-13 months. Of 722 patients with lung cancer, a total of 526 participants were included following the exclusion of 35 participants diagnosed more than 13 months prior to the study and 161 participants who had died during the survey period.* |
| The authors could explore further the limitations regarding the “sample size” representativeness and any biases related to the willingness to participate in the survey. (page 18) | Thank you for these comments. We have added the additional limitations to the Limitations section of the Discussion.  [Discussion – Limitations] Page 20, Lines 398-401  *Second, while the sample size was large enough to conduct a LCPA, there may be potential limitations when it comes to the representativeness of the sample. Additionally, due to the self-reported nature of the data, it is possible that some biases were introduced in terms of participatory willingness to complete the surveys.* |
