# Supplementary material for: Symptom profiles in lung cancer survivors: A latent class approach
Source: PLoS One. 2025 Oct 31;20(10):e0309272. doi: 10.1371/journal.pone.0309272 (PMC12578159; doi:10.1371/journal.pone.0309272)
Supplement: Attachment — Submitted filename: Plos One review.docx [file pone.0309272.s002.docx]

This paper provides an analysis of data on a pre-specified psychoneurological symptom cluster (i.e., pain, fatigue, sleep disturbance, and depression) in patients with lung cancer. The paper takes advantage of an existing database (which is a strength of this study). However, several areas warrant consideration and revision.

1.	This paper is not a symptom cluster study. The author selected a prespecified symptom	cluster. Based on this pre-specified symptom cluster, they used latent variable modeling	to identify patients at increased risk for a higher symptom burden (i.e., symptom	profiles). This conceptualization needs to be made perfectly clear in the Introduction and	Discussion sections of the paper.

2.	The prespecified symptom cluster that was selected in found in the literature and often	labeled a psychoneurological symptom cluster. The published literature on this cluster is	not included in the Introduction. In addition, the findings from previous studies that used	latent variable modeling or cluster analysis to identify subgroups of patients with distinct	symptom profiles are not used in the Discussion to frame the findings from this study.

3.	The authors stated that the did a latent class profile analysis – this statement is not	correct. A latent class analysis uses categorical data to create the subgroups or profiles	of patients. A latent profile analysis uses continuous scores. A latent class profile	analysis uses categorical data and continuous data in the same analysis. This paper	used a latent profile analysis.

4.	Table 1 is not appropriate for this paper. What is needed is a Table that compares the 4	classes on demographic and clinical characteristics. This table should include the results	of the bivariate analyses with statistics and post hoc contrasts. These results define the	risk factors for membership in the different classes.

5.	The multinomial logistic regression analysis is not clinically meaningful. It should be	deleted from the paper.

6.	Use the clinically relevant names of the classes throughout the paper. The class	numbers have no meaning.

7.	The discussion needs to be completely re-written to discuss the classes and associated	risk factors.

8.	Some of these references and countless others need to be reviewed before rewriting the	Introduction and Discussion sections of the paper.

1: Pud D, Ben Ami S, Cooper BA, Aouizerat BE, Cohen D, Radiano R, Naveh P,

Nikkhou-Abeles R, Hagbi V, Kachta O, Yaffe A, Miaskowski C. The symptom

experience of oncology outpatients has a different impact on quality-of-life

outcomes. J Pain Symptom Manage. 2008 Feb;35(2):162-70. doi:

10.1016/j.jpainsymman.2007.03.010. Epub 2007 Dec 21. PMID: 18082357.

2: Miaskowski C, Cooper BA, Paul SM, Dodd M, Lee K, Aouizerat BE, West C, Cho M,

Bank A. Subgroups of patients with cancer with different symptom experiences and

quality-of-life outcomes: a cluster analysis. Oncol Nurs Forum. 2006 Sep

1;33(5):E79-89. doi: 10.1188/06.ONF.E79-E89. PMID: 16955115.

3: Harris CS, Dodd M, Kober KM, Dhruva AA, Hammer MJ, Conley YP, Miaskowski CA.

Advances in Conceptual and Methodological Issues in Symptom Cluster Research: A

20-Year Perspective. ANS Adv Nurs Sci. 2022 Oct-Dec 01;45(4):309-322. doi:

10.1097/ANS.0000000000000423. Epub 2022 Apr 28. PMID: 35502915; PMCID:

PMC9616968.

4: Kim HJ, Barsevick AM, Fang CY, Miaskowski C. Common biological pathways

underlying the psychoneurological symptom cluster in cancer patients. Cancer

Nurs. 2012 Nov-Dec;35(6):E1-E20. doi: 10.1097/NCC.0b013e318233a811. PMID:

22228391.

5: Kim HJ, Malone PS. Roles of biological and psychosocial factors in

experiencing a psychoneurological symptom cluster in cancer patients. Eur J

Oncol Nurs. 2019 Oct;42:97-102. doi: 10.1016/j.ejon.2019.08.005. Epub 2019 Aug

15. PMID: 31479847.

6: George MA, Lustberg MB, Orchard TS. Psychoneurological symptom cluster in

breast cancer: the role of inflammation and diet. Breast Cancer Res Treat. 2020

Nov;184(1):1-9. doi: 10.1007/s10549-020-05808-x. Epub 2020 Aug 15. PMID:

32803635.
