# Supplementary material for: Symptom profiles in lung cancer survivors: A latent class approach
Source: PLoS One. 2025 Oct 31;20(10):e0309272. doi: 10.1371/journal.pone.0309272 (PMC12578159; doi:10.1371/journal.pone.0309272)
Supplement: Attachment — Submitted filename: Responses to Reviewers_Lee_27SEP25.docx [file pone.0309272.s004.docx]

**Date**:	September 27, 2025

**To**:	Dr. Emily Chenette

Editor-in-Chief of *PLOS One*

**From**:	Lena J. Lee, PhD RN

National Institutes of Health, Clinical Center

**Subject**: Submission of manuscript “Symptom Profiles in Lung Cancer Survivors: A Latent Class Approach” requested revisions.

Please find our revised submission of the manuscript entitled “Symptom Profiles in Lung Cancer Survivors: A Latent Class Approach." We provided our responses to the reviewers’ comments and marked our changes in the body of the manuscript with the yellow-colored font. We confirm that this study has passed Ethical Committee Review. We appreciate the thoughtful and thorough critique by the reviewers and believe this has strengthened the quality of our manuscript.

**Comments from the Reviewers**

|  | **Comments** | **Responses to General Comments** |
| --- | --- | --- |
| 1 | This paper is not a symptom cluster study. The author selected a prespecified symptom cluster. Based on this pre-specified symptom cluster, they used latent variable modeling to identify patients at increased risk for a higher symptom burden (i.e., symptom profiles). This conceptualization needs to be made perfectly clear in the Introduction and Discussion sections of the paper. | Thank you for the comments. To address the reviewer’s concern, we clarified the conceptualization of this study.  [Introduction] Page 3, Lines 71-77  *Symptom clusters have been identified using two main approaches: variable-centered approaches and patient-centered approaches [10]. Variable-centered approaches identify symptoms that empirically cluster together though analytic methods (e.g., factor analysis), which create distinct groups of related symptoms (i.e., symptom clusters). Patient-centered approaches (e.g., latent class analysis) identify subgroups of patients with distinct symptom profiles based on one or more symptoms or a pre-specified symptom cluster (e.g., pain, fatigue, sleep disturbance, depression).* |
| 2 | The prespecified symptom cluster that was selected in found in the literature and often labeled a psychoneurological symptom cluster. The published literature on this cluster is not included in the Introduction. In addition, the findings from previous studies that used latent variable modeling or cluster analysis to identify subgroups of patients with distinct symptom profiles are not used in the Discussion to frame the findings from this study. | Thank you for the comments. We added the following language regarding our pre-specified psychoneurological symptom cluster in the Introduction. In addition, we incorporated previous studies that used latent variable modeling and cluster analysis, and we discussed them in the Discussion section.  [Introduction] Page 4, Lines 81-87  *Building on this framework, growing evidence suggests that psychoneurological symptoms (e.g., pain, fatigue, sleep disturbance, depression) often co-occur, forming a cluster that may reflect underlying psychological and/or neurological dysfunction and is frequently observed among cancer patients [13].*  [Discussion] Page 17, Lines 296-302  *Despite these variations, two common subgroups have been consistently observed across studies that examined the same pre-specified psychoneurological symptom cluster as in our study (pain, fatigue, sleep disturbance, and depression) in diverse oncology populations, including lung cancer patients, using cluster analysis or LPA: an all low subgroup (within normal limits) and an all high subgroup [13,28,31-33]. Building on this evidence, our study further highlights the prevalence of these two common subgroups (all low and all high) among lung cancer survivors.* |
| 3 | The authors stated that the did a latent class profile analysis – this statement is not correct. A latent class analysis uses categorical data to create the subgroups or profiles of patients. A latent profile analysis uses continuous scores. A latent class profile analysis uses categorical data and continuous data in the same analysis. This paper used a latent profile analysis. | Thank you for the clarification. As per your suggestion, we have revised the term to “latent profile analysis” throughout the manuscript. |
| 4 | Table 1 is not appropriate for this paper. What is needed is a Table that compares the 4 classes on demographic and clinical characteristics. This table should include the results of the bivariate analyses with statistics and post hoc contrasts. These results define the risk factors for membership in the different classes. | Thank you very much for your thoughtful comments. To address the reviewer’s concern, we have revised Table 1 to compare the classes on demographic and clinical characteristics. After careful discussion with our statistician, who is also one of our co-authors, we conducted multinomial logistic regression to investigate the risk factors associated with class membership, as specified in our study objectives. We believe this approach appropriately identifies the factors influencing membership in the different classes. |
| 5 | The multinomial logistic regression analysis is not clinically meaningful. It should be deleted from the paper. | We sincerely appreciate the reviewer’s thoughtful comments. As above mentioned, following thorough consideration, we decided to retain this approach, as it allows us to define relevant risk factors while also contributing to the future predictability of the identified profiles. |
| 6 | Use the clinically relevant names of the classes throughout the paper. The class numbers have no meaning. | Thank you for your comments. To address the reviewer’s concern, we revised names of the classes throughout the manuscript. |
| 7 | The discussion needs to be completely re-written to discuss the classes and associated risk factors. | Thank you very much for your thoughtful comments. Since no changes were observed in the associated risk factors, we did not revise that specific section of the Discussion; however, we have expanded the Discussion overall to provide greater clarity and context. |
| 8 | Some of these references and countless others need to be reviewed before rewriting the Introduction and Discussion sections of the paper. | Thank you for the thoughtful suggestions. We added the suggested citations and incorporated them into the manuscript. |
